# Periodontal Status in Smokers and Nonsmokers: A Clinical, Microbiological, and Histopathological Study

**DOI:** 10.1155/2012/571590

**Published:** 2012-02-14

**Authors:** Maddipati Sreedevi, Alampalli Ramesh, Chini Dwarakanath

**Affiliations:** ^1^Indira Gandhi Institute of Dental Sciences, Pondicherry 607402, India; ^2^The Oxford College of Dental Sciences, Bangalore 560068, India

## Abstract

A case-control study was done to assess the influence of smoking on clinical, microbiological, and histopathological parameters. *Methods.* Two hundred dentate male patients (100 smokers and 100 nonsmokers) ranging between 25 and 50 years were enrolled in the study. Periodontal parameters were recorded. Plaque samples were collected for microbial analysis for BANA test. Gingival biopsies were obtained from selected site for assessing histopathological changes. *Results.* Both groups showed almost similar plaque levels (*P=0.258*), but smokers had reduced gingival (0.62 ± 0.31) and bleeding indices (28.53 ± 17.52) and an increased calculus index (1.62 ± 0.36). Smokers had an increased probing depth of 4–7 mm (*P=0.009*) and overall increased CAL. No difference in microbiota was found between the two groups. Histopathologically smokers showed a decreased blood vessel density (8.84 ± 0.96) and inflammatory cells (52.00 ± 9.79). *Conclusions.* It is quite possible that many of the pathogenic mechanisms involved in tissue degradation in periodontitis in smokers could be quite different from those in nonsmokers.

## 1. Introduction

Periodontitis is defined as an inflammatory disease of the supporting tissues of the teeth caused by specific microorganisms or groups of specific microorganisms, resulting in progressive destruction of the periodontal ligament and alveolar bone with pocket formation, recession, or both [1].

Periodontal diseases are infections caused by dental plaque, but risk factors can modify the host response to microbial aggression [2]. Some of the known risk factors are diabetes, tobacco smoking, pathogenic bacteria, and microbial tooth deposits.

 Smoking is a known risk factor for many diseases, and increasing evidence suggests that smoking adversely affects periodontal health [3]. The concept that smoking tobacco may be detrimental to periodontal health is not new. In fact, Pindborg observed an association between acute necrotizing ulcerative gingivitis and smoking nearly 60 years ago [4]. Since then, various investigators have attempted to identify the role of tobacco smoking in the etiology of periodontal diseases. These studies suggest that smoking is a single, modifiable environmental risk factor responsible for excess prevalence of periodontal disease in the population and has a direct influence on periodontal variables.

The 1996 World Workshop in Periodontics reviewed a number of studies and confirmed that “smoking entailed an overall increased risk for severe periodontal disease and estimated overall odds ratio 2.82” [5].

Earlier investigators had attributed the increased prevalence and severity of periodontal disease seen in smokers to the greater presence of plaque and calculus than compared to nonsmokers. However, with the better understanding of the host response, evidence suggests that the effect of smoking on periodontal status is independent from the plaque index and oral hygiene of individual. So, this clearly suggests that smoking has a direct influence on periodontal tissues.

Smokers have been associated with deeper pockets and greater attachment loss, more pronounced radiographic evidence of furcation involvement, and increased alveolar bone loss. There is an established biologic rationale for the negative effect of smoking on periodontal tissues. It has an immunosuppressive effect on the host, adversely affecting host-bacterial interactions, and this alteration may be due to changes in the composition of subgingival plaque. Smoking may also provide a conducive environment for some of the periodontopathic species in the plaque and may be one reason why smoking is a risk factor in periodontal disease development [6].

 Smoking exerts a strong, chronic, and dose-dependent suppressive effect on gingival bleeding on probing. Bleeding on probing was less evident in smokers than nonsmokers, indicating its effect on gingival blood vessels. The mechanism by which smoking suppresses gingival bleeding is not understood exactly [7].

On the basis of the observation that smokers may present with a lower level of gingival inflammation, it has been speculated that the gingival blood flow in smokers may be less in comparison to nonsmokers. This would also induce a decreased local host response. So, smoking is thought mainly to affect the periodontal tissues by way of the vascular and immunological response of the body.

While there is overwhelming clinical evidence to associate smoking with destructive periodontal disease, the mechanisms that may predispose smokers to periodontitis remain to be fully elucidated.

To explore more into the above facts, this study evaluated the clinical, microbiological and histopathologic changes in smokers and compared these to the nonsmokers.

## 2. Materials and Methods

### 2.1. The Study Population

Two hundred dentate male patients comprising hundred smokers and hundred nonsmokers all in the age group ranging between 25–50 years were selected from among the patients referred to the Department of Periodontics at The Oxford Dental College Hospital and Research Centre, Bangalore. The subjects for the study were selected randomly taking into consideration only their smoking history.

Ethical clearance for the study was obtained from the Research and Ethical Committee of The Oxford Dental College Hospital and Research center, Bangalore.

### 2.2. Selection of Subjects

The following criteria were applied while selecting patients under smokers group (test group):

1. Patient should have been smoking since three years or more.
2. Patient should not have had any known systemic conditions that could influence periodontal health.
3. Patient should not have been subjected to periodontal therapy or any antibiotic medication during the last 6 months.

The criteria for choosing patients under nonsmokers group (Control group) were as follows:

1. Subjects should not have smoked at anytime in their lives.
2. Patient should not have had any known systemic conditions that could influence periodontal health.
3. Patient should not have been subjected to periodontal therapy or any antibiotic medication during the last 6 months.

Exclusion criteria for both groups include female patients, former smokers, and aggressive periodontitis patients.

All the patients were subjected to a detailed case history. The following data was also obtained from subjects belonging to smokers group:

1. Number of cigarettes or beedies consumed daily.
2. Frequency of smoking.
3. Number of years of smoking.

Informed consent was obtained from all the subjects.

A thorough periodontal examination was carried out under good artificial light, and parameters selected for the study were carefully recorded. However, the consistency and accuracy of measurement were randomly checked by another examiner so as to keep interexaminer variation negligible.

Plaque index of Silness and Loe [8], gingival index of Loe and Silness [9], bleeding index [10], and NIDR calculus index [11] were initially recorded.

### 2.3. Periodontal Status

Parameters such as pocket depth, clinical attachment loss, mobility, and furcation were recorded. The periodontal pocket depth and clinical attachment loss were recorded using a William's periodontal probe. Furcations were assessed using Nabers probe.

Periodontal disease has often been described as site specific. Since the mean scores may not reflect the severity of the problem clearly, it was decided to classify the probing depth sites into three groups as follows:

sites showing <4 mm of probing depth,
sites showing 4–7 mm of probing depth,
sites showing >7 mm of probing depth.

Clinical attachment was classified under three groups as follows:

sites showing attachment loss <4 mm,
sites showing attachment loss between 4–7 mm,
sites showing attachment loss >7 mm.

Numbers of teeth showing mobility, recession, and teeth with furcation were calculated separately.

### 2.4. Microbiological Examination

BANA test is done to assess the microbiological status.

One site with the deepest probing depth is chosen for plaque collection. Supragingival plaque in this site was carefully removed, and then, a curette is used to collect subgingival plaque. The adherent plaque from the curette is wiped onto the BANA-impregnated strip found at the lower edge of the BANA reagent card. An upper reagent strip containing Evan's black dye was then activated through dampening with distilled water, and the strip was folded at the perforation mark so that the lower and upper reagent strips come in direct contact with each other. After folding, the card was inserted into a slot on top of the BANA incubator and incubated for 5 min at 35°C. The light would operate during incubation and would automatically shut off once the heating cycle was completed. A bell sound was heard once the heating cycle is complete. Naphthylamide released due to the presence of any one of the BANA-hydrolyzing bacterial species diffused into the upper reagent strip, where it reacted with the Evan's black dye to form a permanent blue-black colour.

The test was considered positive if blue colour was visible on the upper reagent strip after incubation and was considered negative if no blue colour was visible.

### 2.5. Histopathological Examination

After the clinical examination and microbiological test, preparation was done to take biopsy of the interdental papilla between the lateral incisor and canine of the left side of the upper arch. Biopsy was obtained by sharp dissection with a Bard Parker blade no. 15 under local anesthesia.

The biopsy specimen was immediately transferred to a bottle containing 50% formo-alcohol (50 mL of 10% formalin and 50 mL of alcohol) and kept for 24 hours for fixation.

Slides were prepared by standard histological technique using haemotoxylin and eosin stains.

All the slides were viewed under compound microscope attached with a micrometer scale at 20x (objective) magnification to which a camera was attached. Four views of each slide was then photographed with the scale adjusted for each photograph. These photographs were then transferred to a computer and were assessed.

Numbers of inflammatory cells and blood vessel density (number of blood vessels) were estimated, and then, diameter of each vessel was measured using calipers. Vessels and cells intersecting the grid lines were excluded.

The diameter of blood vessels measured was then divided into three groups as:

Diameter <4 mm; 4–8 mm and >8 mm.

All the observations made were recorded on tables as per the criteria laid out and the data thus collected was subjected to extensive statistical analysis.

### 2.6. Statistical Methods [12, 13]

Chi-square and Fisher exact test have been used to test the proportions of study parameters between nonsmokers and smokers. Students *t* test (two tailed, independent) and Mann-Whitney *U* test have been used to find the significance of study parameters between nonsmokers and smokers. Analysis of variance has been used to find the significant association of pack years and the study parameters. Kruskal-Wallis test (a nonparametric) has been used to find the significant association of PD and CAL with pack years.

The statistical software, namely, SPSS 11.0 and Systat 8.0 were used for the analysis of the data and Microsoft word and Excel have been used to generate graphs, tables, and so forth.

## 3. Results

The mean age of nonsmokers was 35.10 ± 7.14 and the mean age of smokers was 35.13 ± 7.05. The age was matched between the two groups (Table 1).

### 3.1. Distribution of Smokers by Pack Years

12 subjects had a smoking history of <1 pack year, 50 subjects had a history of smoking for about 1–5 pack years, 24 of them for about 5–15 pack years, and 14 of them had a smoking history of >15 years (Table 2).

### 3.2. Periodontal Parameters

Smokers had a slightly higher plaque index than that of nonsmokers. The mean plaque index in smokers was 1.11 ± 0.44, whereas in nonsmokers it was 1.04 ± 0.44 with *P* value of 0.258. This difference was not statistically significant as shown in Table 3.

Nonsmokers had a higher gingival index score of 0.86 ± 0.41 than smokers who had a mean score of 0.62 ± 0.31. The difference was found to be statistically significant (*P* ≤ 0.001) (Table 3).

Smokers demonstrated lower bleeding scores than nonsmokers. The mean bleeding index in nonsmokers was 39.54 ± 23.03 and of smokers was 28.53 ± 17.52. This difference was statistically significant (*P* = 0.001) (Table 3).

Smokers demonstrated consistently higher scores for calculus than nonsmokers. The mean calculus index in smokers was 1.62 ± 0.36 as compared to 1.40 ± 0.55 in nonsmokers. This difference showed statistical difference between the two groups (*P* = 0.001) (Table 3).

Comparison of PD between nonsmokers and smokers showed that 98.57% of sites in nonsmoker group had probing depth <4 mm, whereas this was 97.22% in smokers group. This was statistically significant with *P* = 0.006.

The percentage of sites showing PD of 4–7 mm was 1.29 in nonsmokers and 2.58 in smokers. Again, this was also statistically significant with *P* value of 0.009.

In both nonsmokers and smokers, few sites showed PD >7 mm which was not statistically significant at *P* = 0.859 (Table 4).

The percentage of sites showing CAL <4 mm in nonsmokers was 3.52 and in smokers was 6.78 with *P* value of 0.004 which was highly significant.

3.29% of sites had CAL of 4–7 mm in nonsmokers, and the same was 7.99% in smokers. This showed a statistically significant result with *P* = 0.004.

The mean percentage of sites in nonsmokers with CAL >7 mm was 0.09, and this value was higher in smokers of about 3.39%, which was again significant with *P* = 0.030.

The above results indicate that there was an increased attachment loss in smokers when compared to nonsmokers (Table 5).

Smokers had significantly more teeth with mobility (29%) when compared to nonsmokers (16.0%) with *P* = 0.030 which was statistically significant (Table 6).

Smokers had significantly more teeth with recession (79.0%) when compared to nonsmokers (59.0%) with *P* = 0.003 which was significant (Table 7).

Smokers had significantly more teeth with furcation (68.0%) when compared to nonsmokers (84.0%) with *P* = 0.013 (Table 8).

### 3.3. Microbiological Test (BANA Test)

The test was positive in 38% of subjects in nonsmokers and 36% of subjects who were smoking. The results showed no difference in microbiota in both the groups with *P* = 0.836 (Table 9).

### 3.4. Histopathological Parameters

Number of inflammatory cells in nonsmokers was 64.70 ± 12.68 and 52.00 ± 9.79 in smokers. Even though smokers had less cells than nonsmokers, this was not statistically significant (*P* = 0.319) (Table 10).

Blood vessel density in nonsmokers was 11.12 ± 1.23, and the density reduced in smokers, where it was 8.84 ± 0.96. This difference was not significant (*P* = 0.179) (Table 10).

### 3.5. Diameter <4 mm

Nonsmokers had 46 vessels with this diameter, whereas they were 44 in smokers.

This showed statistically similar results between the two groups (*P* > 0.05) (Table 11).

### 3.6. Diameter 4–8 mm

Vessels with this diameter were 46 in nonsmokers and 39 in smokers. So, blood vessel diameter 4–8 mm was significantly less in smokers (*P* = 0.073) (Table 12).

### 3.7. Diameter >8 mm

Vessels with this diameter were significantly similar in both the groups, as both the groups had 10 vessels in this category (*P* > 0.05) (Table 13).

 The periodontal parameters in smokers were then correlated with the number of pack years to evaluate the effects of chronic smoking.

 Plaque index increased significantly with the increase in number of pack years (*P* = 0.002) (Table 14).

Gingival index did show a decrease as pack years increased to 15 years but later increased as pack years increased to more than 15 years with (*P* = 0.577) (Table 14).

Bleeding index decreased up to 5–15 years and then showed an increase when the pack years was >15 years (*P* = 0.237) (Table 14).

Calculus index showed an increased value as pack years increased, which is suggestive of significance (*P* = 0.058) (Table 14).

Probing depth <4 mm significantly decreased as pack years increased with *P* value of <0.001.

Probing depth of 4–8 mm significantly increased as pack years increased with *P* value of <0.001 (Table 15).

Clinical attachment loss <4 mm, 4–8 mm and >8 mm significantly increased as pack years increased with *P* = 0.005, <0.001 and <0.001, respectively (Table 15). See Figures from 1 to 6 for further clarification.

## 4. Discussion 

The global rise in the number of people addicted to smoking, and mortality and morbidity associated with it, has made smoking a major public health hazard. But the exact mechanism how smoking increases the severity for periodontitis is not fully understood. Whether smoking causes a local effect on the periodontium or the systemic effects of smoking that causes periodontal disease is not known. This study was done to know the effects of smoking on the periodontium by studying the clinical, microbial, and histopathological parameters.

To equate and nullify the effect of all other possible contributing factors, patients belonging to same age group (25–50 yrs) with no other known systemic problems were selected for the study. Although some of the previous studies [3, 7, 14, 15] included subjects who had quit smoking for a period of 2–5 yrs or more under the nonsmokers category, it was decided in this study to exclude former smokers in order to eliminate any long-term effect of smoking on periodontal tissues.

 Females were purposely excluded from the study for the main purpose that it would have been difficult to recruit females who admit that they smoke. The other reason for excluding them was to avoid potential hormone-induced microcirculatory changes [16–18].

Since patients with any known systemic problems were not included, it was considered reasonable that comparisons made between smokers and nonsmokers group accurately reflected the influence of smoking on periodontium.

Patients were selected only on the basis of their smoking status and not depending on their periodontal status, as some previous studies [2, 19–21] selected patients who were diagnosed with periodontitis. This selection was mainly done as it was decided to study the relationship of smoking to periodontal health. The duration of smoking in the cases ranged from 3 to 30 yrs, which was later calculated into pack years.

The oral hygiene status as depicted by plaque scores were almost similar in both the groups even though smokers had slightly higher scores that were not significant and this finding is in agreement with the other previous studies [2, 3, 22–24]. Contradicting these studies, others have shown higher plaque levels in smokers [25–31]. Few studies even showed less plaque levels in smokers [32, 33]. One study concluded that poorer cleanliness found in smokers both before and after toothbrushing may be explained, in part at least, by their shorter tooth brushing time [34].

There was significantly less gingival inflammation in smokers, which is in agreement with the earlier studies [22, 32, 35–37].

Suppression in smokers of the normally developing gingival inflammatory reaction associated with plaque provocation may be due to tobacco smoke products interfering with the vascular inflammatory response. It is generally accepted that smoking causes vasoconstriction of peripheral vessels. It is therefore conceivable that such a constrictive action on gingival vessels would result in the suppression of vascular properties of inflammation such as bleeding, redness, and exudation. Smoking has previously been shown to affect oral PMN leukocytes, indicating an impairment of PMN-function [38, 39]. Thus, smoking seems to influence both vascular and cellular properties of the inflammatory reaction. The suppression of vascular inflammatory reaction under the influence of smoking might then indicate an impairment of the defense mechanisms within the tissues and possibly render them more susceptible to plaque infection.

These results are not in agreement with other studies [40–42], showing increased inflammation. Some studies showed no differences in the inflammatory status between smokers and nonsmoker [32, 43].

If, however, it is hypothesized that the inflammatory response of the gingiva is a clinical manifestation of the degree or capacity of the host to respond to irritation (a postulate which has also been proposed by others (Page) [44], then the present findings may be explained in the following manner. In smokers, what has been termed the lowered incidence rate may, perhaps, be better understood as a reflection of a reduction of the capacity of the host to mount an effective defence through the inflammatory process.

Another not necessarily contradictory hypothesis may be advanced to explain these findings. It is possible that substances in tobacco smoke can reduce the capacity of microorganisms in plaque to produce irritants. Consequently, the extent to which gingival inflammation occurs, and the extent to which it is necessary for the host to maintain an inflammatory response, might be less marked, yielding a lowered incidence rate [37]. 

Decreased inflammatory signs in the present study can be attributed to decrease in the number of inflammatory cells as shown histopathologically. Smoking can drastically alter the typical presentation of gingivitis and periodontitis by masking the signs of inflammation. Thus, the diagnosis of these diseases is made more difficult, yet the disease effects are worse.

Regarding bleeding on probing, this study is in agreement with other studies [33, 45–47] showing decreased gingival bleeding in smokers. The mechanisms by which smoking suppresses gingival bleeding are not understood. Traditionally, the reduced bleeding in smokers has been attributed to gingival vasoconstriction induced by the actions of nicotine-stimulated adrenaline and noradrenaline on *α*_1_-adrenergic receptors. There is some evidence to support this theory in animal models. However, the available evidence support this hypothesis in humans is not concluded, as smoking can cause vasodilatation in some tissues. Contradicting these results, some studies have shown increased gingival bleeding [3, 48, 49].

The results of the present study showed more calculus deposition in smokers agreeing with the results of the other studies [29, 41, 48, 50]. It seems likely, therefore, that smoking primarily affects the mineralization rate rather than the formation rate of supragingival plaque. The reason why smoking is associated with an elevated risk for supragingival calculus deposition remains incompletely understood. It may exert its influence systemically via the saliva or locally via a conditioning of tooth surfaces to deposition, or both. It may be speculated that smoking causes a modification of the saliva resulting in elevated levels of calcium and possibly phosphorous. Further, it can be considered that a reduced flow may cause elevation of the calcium and phosphate concentrations. Thus, paradoxically, smoking might promote the calcification of subgingival plaque notwithstanding its suppressive action on some inflammatory events. This may have accounted for greater calculus scores in smokers [51, 52].

Chen et al. have shown no difference in the calculus deposition in their 10-year longitudinal study [53].

There were no significant difference in periopathogens in this study as confirmed by BANA although this contradicts the study by Kazor et al. [54] who showed a positive relation between BANA pathogens and smoking. This difference could be attributed to 4-plaque samples examined by Kazor et al., but in the present study, only one plaque sample from deepest pocket depth irrespective of diseases activity was considered. This is in agreement with the other earlier studies showing no difference [55–59].

Few other studies showed difference in the subgingival microflora [18, 43, 60, 61], but the microbial analysis varied.

There are problems associated with microbiological investigations of the oral flora that may affect the interpretation of the results of the studies. Sampling methods vary widely, and, together with undoubted differences from site to site within the mouth, such variations may affect the results of studies. Identification of organisms by different methods such as culture-, immunofluorescence-, and DNA-based techniques gives rise to potentially different outcomes. Under these circumstances, it was imperative that studies with adequate numbers of subjects were performed in order to overcome the background of extreme variation, which will potentially mask the effects of smoking on the oral microflora. Of those that appear to satisfy these requirements, some early studies tended to show no differences. However, there are now a number of studies that suggest a trend for smokers to harbour more or greater numbers of potential periodontalpathogens than nonsmokers without increasing the amount of plaque. This undoubtedly supports the attractive hypothesis that a significantly different subgingival environment in smokers, related to an altered immune response, should result in a different microflora. Further investigation with the latest methods is still required to confirm that such differences are directly related to smoking.

Smokers showed a reduced blood vessel density compared to nonsmokers contradicting other studies [62–64] who showed no difference. The suppressive effect on the vasculature can be observed through less gingival redness, lower bleeding on probing, and fewer vessels visible clinically and histologically.

Blood vessel diameter showed no difference in blood vessels with diameter of <4 mm and >8 mm. Only the in-between categories 4–8 mm significantly were less in smokers, explaining the vasoconstrictive effect of smoking on few blood vessels. While it is difficult to compare the results of Baab and Oberg [65], Meekin et al. [66], and Mavropoulos et al. [67] to the present study because of the variations in study design, including the smoking habits and other characteristics of the study population, the methods employed to analyse the data, and the lack of control group in studies by Baab and Oberg [65] and Mavropoulos et al. [67], it would be difficult to conclude that smoking causes gingival vasoconstriction.

Smokers had significantly more sites with probing depths 4–7 mm. Other studies have also shown deeper pockets [2, 3, 24, 26, 30, 33].

The present study showed increased attachment loss and this increased as the years of smoking increased. Few other studies showed the same results [2, 30, 53].

Studies by Kamma et al. [21], Calsina et al. [2], and Gunsolley et al. [68] are in agreement with the present study showing increased gingival recession in smokers, whereas the study by Muller et al. [69] found no difference and did not support the hypothesis that smokers have more gingival recession. 

Smokers had more teeth furcation involvement and mobility, and this have been earlier confirmed by Kerdovongbundit and Wikesjo [70],Bergström [71], and Calsina et al. [2].

The present study showed that smokers have more severe periodontal disease than non-smokers and that it has a strong, chronic, dose-dependent effect on periodontium.

Tobacco smoking, mostly in the form of cigarette smoking, was recognized as the important environmental risk factor in periodontitis. Tobacco smoking affects the oral environment and ecology, the gingival tissues and vasculature, the inflammatory response, the immune response, and the homeostasis and healing potential of the periodontal connective tissues. While there is overwhelming clinical evidence to associate smoking with destructive periodontal disease, the mechanisms that may predispose smokers to periodontitis remain to be fully elucidated. It was also apparent that while smoking may suppress gingival angiogenesis, the mechanisms by which cigarette smoking dampens periodontal inflammation are not yet fully understood.

The findings of the present study emphasizes that periodontal tissue in smokers are affected more than the controls with minimal signs of inflammation. The best way to prevent periodontitis in smokers is by enrolling the subjects in smoking cessation programmes, and so, it is an obvious implication that smoking prevention should be included in dental public health education by advocating, advising, and facilitating smoking cessation programmes among the patients.

## Figures and Tables

**Figure 1:**
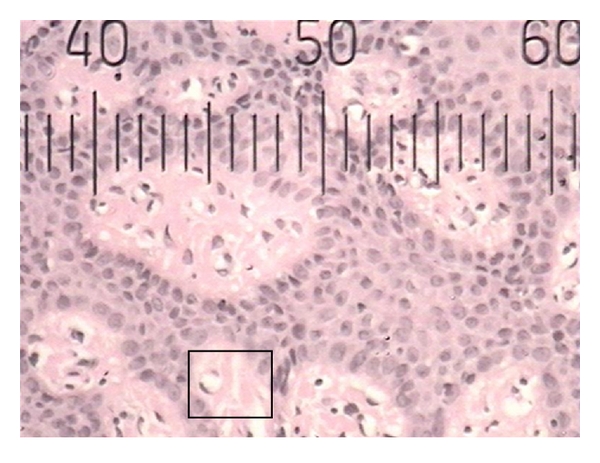
Histopathological photograph of gingival tissue in smoker showing smaller blood vessels.

**Figure 2:**
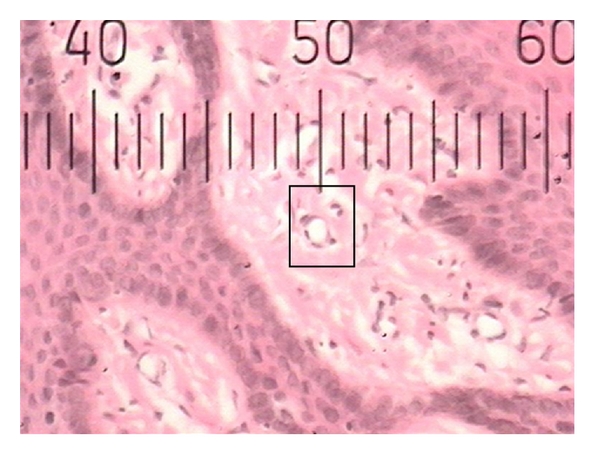
Histopathological photograph of gingival tissue in nonsmoker showing smaller blood vessels.

**Figure 3:**
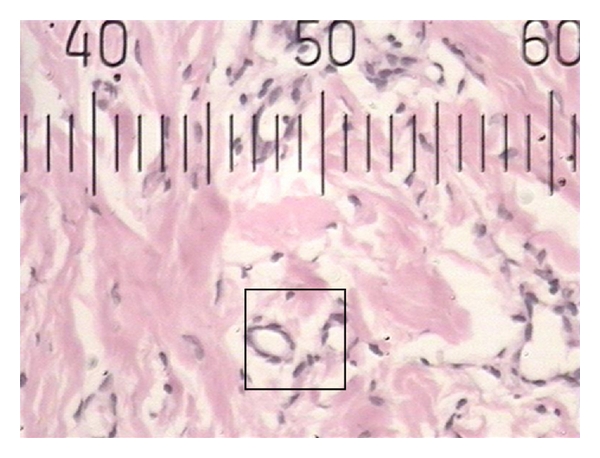
Histopathological photograph of gingival tissue in smoker showing larger blood vessels.

**Figure 4:**
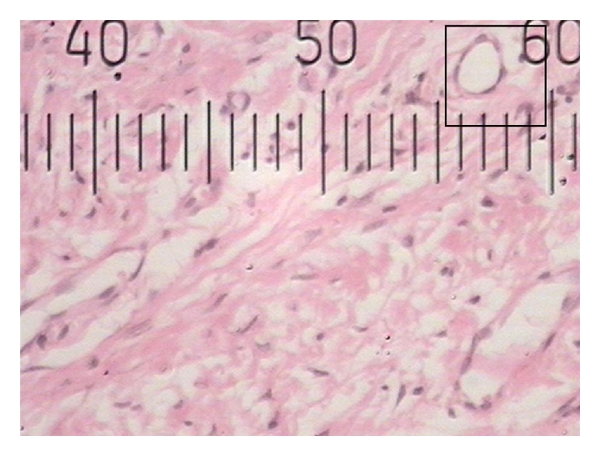
Histopathological photograph of gingival tissue in nonsmoker showing larger blood vessels.

**Figure 5:**
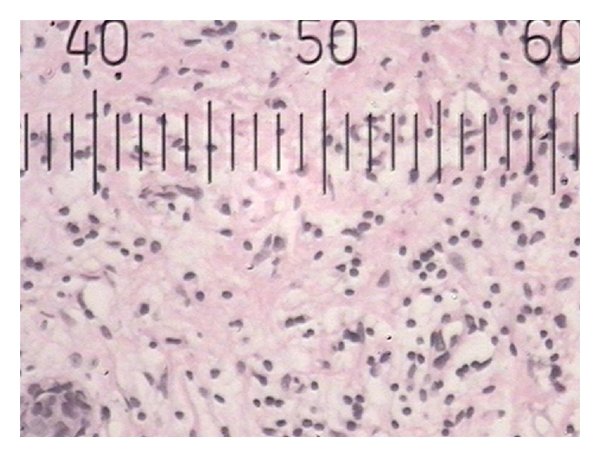
Histopathological photograph of gingival tissue in smoker showing inflammatory cells.

**Figure 6:**
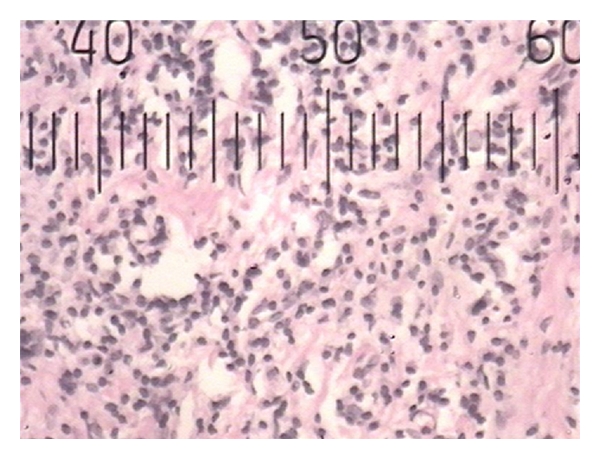
Histopathological photograph of gingival tissue in nonsmoker showing inflammatory cells.

**Table 1:** Age distribution.

Age in years	Nonsmokers		Smokers	
	No	%	No	%
25–30	36	36.0	38	38.0
31–35	20	20.0	16	16.0
36–40	22	22.0	23	23.0
41–45	11	11.0	15	15.0
46–50	11	11.0	8	8.0

Total	100	100.0	100	100.0

Mean ± SD	35.10 ± 7.14		35.13 ± 7.05	

Remarks	Samples are age matched with *P* = 0.976			

**Table 2:** Distribution of pack years.

Pack years	Number (*n* = 100)	%
<1 year	12	12.0
1–5 years	50	50.0
5–15 years	24	24.0
>15 years	14	14.0

**Table 3:** Comparison of PI, GI, BI, and CI between nonsmokers and smokers. Results are presented in Mean ± SD (min-max).

Parameters	Nonsmokers	Smokers	*P* value
PI	1.04 ± 0.44 (0.33–2.20)	1.11 ± 0.44 (0.40–2.31)	0.258

GI	0.86 ± 0.41 (0.16–2.00)	0.62 ± 0.31 (0.12–1.54)	<0.001**

BI	39.54 ± 23.03 (2.23–100)	28.53 ± 17.52 (3.30–100.0)	0.001**

CI	1.40 ± 0.55 (1.40–2.00)	1.62 ± 0.36 (0.63–2.00)	0.001**

**Table 4:** Comparison of probing depth between nonsmokers and smokers. Results are presented in trimean (min-max).

Parameters	Nonsmokers	Smokers	*P* value
PD <4 mm%	98.57 (13.33–100)	97.22 (0–100)	0.006**

PD 4–7 mm%	1.29 (0–70.56)	2.58 (0–76.92)	0.009**

PD >7 mm %	0 (0–16.11)	0 (0–12)	0.859

**Table 5:** Comparison of clinical attachment loss between nonsmokers and smokers. Results are presented in trimean (min-max).

Parameters	Nonsmokers	Smokers	*P* value
CAL <4 mm%	3.52 (0–49.46)	6.78 (0–37.10)	0.004**

CAL 4–7 mm%	3.29 (0–72.58)	7.99 (0–83.97)	0.004**

CAL >7 mm%	0.09 (0–53.3)	3.39 (0–54.49)	0.030*

**Table 6:** Comparison of mobility between nonsmokers and smokers.

Number of teeth with mobility	Nonsmokers (*n* = 100)	Smokers (*n* = 100)
	16%	29%

**Table 7:** Comparison of recession between nonsmokers and smokers.

Number of teeth with recession	Nonsmokers (*n* = 100)	Smokers (*n* = 100)
	59%	79%

**Table 8:** Comparison of furcation between nonsmokers and smokers.

Number of teeth with Furcation	Nonsmokers (*n* = 100)	Smokers (*n* = 100)
Present	16%	32%

**Table 9:** Comparison of BANA test positive between nonsmokers and smokers.

BANA test results	Nonsmokers (*n* = 50)	Smokers (*n* = 50)
Positive	19 (38.0%)	18 (36.0%)

Negative	31 (62.0%)	32 (64.0%)

**Table 10:** Comparison of number of IC and BV density between nonsmokers and smokers. Results are presented in Mean ± SD (min-max).

Number of IC and BV density	Nonsmokers (*n* = 50)	Smokers (*n* = 50)	*P* value
Number of IC	64.70 ± 12.68 (4–468)	52.00 ± 9.79 (3–297)	0.319

BV density	11.12 ± 1.23 (2–45)	8.84 ± 0.96 (1–27)	0.179

**Table 11:** Comparison of BV diameter <4 between nonsmokers and smokers.

BV diameter <4 mm	Nonsmokers (*n* = 50)	Smokers (*n* = 50)
0	4 (8.0%)	6 (12.0%)
1–10	41 (82.0%)	43 (86.0%)
>10	5 (10.0%)	1 (2.0%)

**Table 12:** Comparison of BV diameter 4–8 mm between nonsmokers and smokers.

BV 4–8 mm	Nonsmokers (*n* = 50)	Smokers (*n* = 50)
0	4 (8.0%)	11 (22.0%)
1–10	37 (74.0%)	35 (70.0%)
>10	9 (18.0%)	4 (8.0%)

**Table 13:** Comparison of BV diameter >8 mm between nonsmokers and smokers.

BV >8	Nonsmokers (*n* = 50)	Smokers (*n* = 50)
0	40 (80.0%)	40 (80.0%)
1–10	10 (20.0%)	10 (20.0%)
>10	—	—

**Table 14:** Mean pattern of clinical parameters with years of smoking.

Clinical parameters	Pack Years of smoking				*P* value
	<1 years	1–5 years	5–15 years	>15 years	
PI	1.05 ± 0.31	0.99 ± 0.35	1.16 ± 0.44	1.47 ± 0.59	0.002**
GI	0.69 ± 0.32	0.60 ± 0.26	0.58 ± 0.31	0.69 ± 0.41	0.577
BI	31.04 ± 15.21	25.75 ± 17.20	28.58 ± 15.33	36.29 ± 22.58	0.237
CI	1.64 ± 0.37	1.54 ± 0.35	1.69 ± 0.39	1.81 ± 0.28	0.058

**Table 15:** Pattern of clinical parameters with years of smoking.

Clinical parameters	Years of smoking				*P* value
	<1 years	1–5 years	5–15 years	>15 years	
PD <4 mm%	99.38 (84.2–100.0)	97.78 (0–100)	96.06 (17.31–100.0)	87.21 (63.33–100)	<0.001**

PD 4–7 mm%	0.62 (0–14.58)	1.66 (0–33.33)	3.81 (0–76.92)	12.9 (0–32.67)	<0.001**

PD > 7 mm%	0 (0–0.52)	0 (0–12.00)	0 (0–6.17)	0.07 (0–4)	0.724

CAL <4 mm%	4.16 (0–12.50)	5.67 (0–37.10)	6.96 (0–36.56)	15.61 (0.64–26.11)	0.005**

CAL 4–7 mm%	2.94 (0–14.06)	3.87 (0–71.88)	10.98 (0–42.98)	38.06 (2.78–83.97)	<0.001**

CAL >7 mm%	0 (0–0)	0 (0–24)	0.23 (0–54.49)	2.25 (0–20.0)	<0.001**
